# Editorial: Understanding mitochondrial dynamics and metabolic plasticity in cancer stem cells: Recent advances in cancer treatment and potential therapeutic approaches

**DOI:** 10.3389/fonc.2023.1155774

**Published:** 2023-03-14

**Authors:** Pramod Darvin, Varun Sasidharan Nair

**Affiliations:** ^1^ Cancer Research Division, Rajiv Gandhi Centre for Biotechnology, Trivandrum, India; ^2^ Department Experimental Immunology, Helmholtz Centre for Infection Research, Braunschweig, Germany

**Keywords:** mitochondrial dynamics, mitophagy, mitochondrial immune evasion, cancer stem cell (CSC), hypoxic microenvironment

Mitochondria and mitochondrial homeostasis have been shown to play critical roles in the pathology of various chronic medical conditions including cancer. In cancer, mitochondria are associated with most of the basic cellular mechanisms and signaling events. Being the powerhouse of the cell, mitochondria control cellular energy metabolism and the generation of ATP through oxidative phosphorylation and reactive oxygen species (ROS). Mitochondria are also responsible for the maintenance of cellular homeostasis through controlled cell death. Several factors contribute to the vulnerability of mitochondrial genes to mutation, including the lack of proof-reading mechanism in the replication process, high ROS levels, and the absence of histone proteins (1, 2). This results in a high degree of acquired heterogeneity in tumor microenvironments during disease progression. Due to the direct involvement of mitochondria in cellular events, mitochondrial genes and mitochondrial proteins have immense potential as therapeutic targets and biomarkers for early detection and prognosis.

The mitochondrial DNA (mtDNA) copy number is a direct indication of mitochondrial activity and is known to be associated with cancer by influencing numerous cellular mechanisms. It is also interesting that both increases and decreases in mtDNA copy number alterations are responsible for cancer initiation and progression (3). In adult gliomas, the mtDNA copy number has been evaluated as an age-related predictive marker (4), and, in colon cancer (CRC), it has been reported as a predictor for poor prognosis (5). Moreover, mtDNA copy number variation has been found to be associated with poor prognosis of various other cancers such as cervical cancer, breast cancer, esophageal squamous carcinoma, and chronic lymphocytic leukemia (6–9). Apart from the CNVs, mitochondrial protein expressions were also reported as prognostic biomarkers. Ubiquinol cytochrome *c* reductase binding protein (UQCRB), the protein responsible for the stabilization of mitochondrial ETC complex-III, was reported as a biomarker for CRC. The overall expression and CNV have been reported to be highly associated with CRC progression (10). In a case-control study of 260 renal cell carcinoma patients and 280 matched control individuals, a decreased mtDNA copy number was reported as a heritable predictive marker for higher cancer incidence (11).

In response to cell demands and environmental conditions, mitochondria can divide (mitochondrial fission) and fuse (mitochondrial fusion) (12). These dynamics have important roles in the pathology of cancer. The balance between fission and fusion is crucial in the maintenance of cellular processes such as energy metabolism, calcium signaling, oxygen sensing, and ROS generation. Most genes regulating mitochondrial fission and fusion are encoded by nuclear genes (13). In solid tumours, mitochondrial fission induces a reduction in the expression of MHC-I, causing immune escape (14) and making it a target to prevent immune evasion. Similarly, in triple negative breast cancer (TNBC) samples, significantly increased mitochondrial fission is associated with poorer survival (15). It is also observed that the survival of TNBC cells increased with a positive feedback loop to mitochondrial fission, by enhancing the Notch- and surviving-mediated pathways (15). On the other hand, Humphries et al. reported that TNBC with increased mitochondrial fission showed reduced metastatic potential (16).

Mitophagy, the elimination of defective mitochondria through autophagy, is considered to be a quality control mechanism, and any defects in mitophagy lead to impairment of mitochondrial functions, creating pathological alterations (17). Dysfunction of mitophagy leads to tumorigenesis (18) and the role of mitophagy varies with tumour progression. In a study using mitochondrial depletion, Yu-Seoun et al., demonstrated that mitochondrial dysfunction can lead to cancer cells acquiring stem cell-like properties (19). Furthermore, the mitochondrial density decreases as a result of increased mitophagy, which results in low reactive oxygen levels and low energy levels in the cells. Consequently, cells that possess stem cell-like quiescence properties survive in hypoxic conditions, resulting in residual cells and relapses of cancer. The majority of chemotherapies target rapidly dividing cells and ROS-producing cells, both of which become ineffective against quiescent cells, resulting in chemo resistance (20).

Metabolic reprogramming is an extremely prevalent feature of cancer cells and is directly linked to mitochondria. Mitochondria are indispensable to cancer cells because of their ability to generate ATP. In breast cancer cells, Lu et al., reported that the overexpression of mitochondrial fission regulator protein (MTFR2) alters the metabolism of glucose. MTFR2 changes oxidative phosphorylation (*OXPHOS*) into glycolysis in a HIF1α- and HIF2α-dependent manner (21). In hypoxic conditions, cancer cells also switch from *OXPHOS* to glycolysis. During this switching, anaerobic glycolysis generates lactate as the end product, which assists the cells in reducing ROS levels by utilizing metabolic intermediates such as pyruvate (22). In addition, it has been reported that glycolytic enzymes are upregulated during hypoxia; therefore, inhibiting these enzymes could be a promising way to eradicate residual cells and cancer stem cells (23–25). In addition to switching to glycolysis, the hypoxic microenvironment activates the pentose phosphate pathway (PPP). PPP activation produces NADPH and facilitates cancer cell survival in hypoxic environments by maintaining ROS homeostasis (26, 27).

Notably, mitochondria are the major source of intracellular ROS for a cell. By the oxidation of nucleotides, increased intracellular ROS directly damages nuclear and mitochondrial DNA. Similarly, reduced ROS levels cause cancer cells to enter a quiescent state, preventing them from being damaged by oxidative stress (28). Low levels of ROS are reported in cancer stem cells and metabolically inactive drug resistance cells (1). It has been shown that mitochondria-targeted photodynamic therapies (PDT) are effective in eliminating quiescent cancer stem cells and chemo-resistant cancers that are in a low-energy state (29). Integrated PDT uses the cancer cell’s ROS and metabolic state to convert prodrugs into active photosensitizers or to specifically target photosensitizers to mitochondria, resulting in effective photosensitizer accumulation (30). Upon exposure to irradiation, mitochondria suffer irreversible damage, resulting in cell death.

In the tumor microenvironment (TME) hypoxia induces acidification with low pH through the accumulation of lactic acid from glycolysis. The low pH alters the expression of multiple genes that promote cancer cell invasion and metastasis, and inhibits immune cell infiltration into the TME (31). Mitochondria-mediated upregulation of carbonic anhydrase enzyme, responsible for cancer cell survival in acidic environments, is a target for inhibiting cancer progression and metastasis (van Gisbergen et al.). Moreover, at an acidic pH, the immune cells lose their ability to counteract cancer cells. According to a recent study by Yi-Ru et al., decreased mitophagy in T cells leads to the accumulation of depolarized mitochondria and terminal exhaustion of T cells. Notably, T cells treated with nicotinamide riboside recovered mitochondrial fitness and became responsive to PD-1 inhibitors, confirming the role of mitochondrial dynamics in T-cell exhaustion (32). In addition, MDSCs infiltrate tumors by secreting chemokines that are regulated by mitochondria through HIF-1α (28). An immune responsive TME results from targeting mitochondria to impede chemokine production, thereby affecting the recruitment of MDSCs and Tregs.

The transfer of mitochondria between cells of the TME is yet another mechanism for cell survival and immune evasion. Saha et al., with the help of advanced technologies, depicted the nanotube-mediated transfer of mitochondria between cancer cells and immune cells. The cancer cells benefit from this exchange by generating more energy and increasing their cell division, whereas the immune cells become inactive and exhausted from this exchange. A promising strategy to target the TME for cancer treatment is to inhibit nanotube formation to enhance immune therapies (33).

Mitochondria provide resistance against chemotherapeutic agents through a number of different ways. As discussed previously, the most important way is ROS homeostasis. Additionally, ATP-dependent efflux pathways for multidrug resistance (MDR) (34), as well as TME acidifications, are reported to have a mechanistic impact on chemoresistance (35). Besides chemoresistance, the ROS scavenging ability protects cancer cells against radiotherapy (27).

Another therapeutic target is mitochondrial biogenesis in cancer cells. In a bioinformatics-based analysis of lung cancer patients, the key gene for mitochondrial biogenesis, HSPD1, was confirmed as a predictive biomarker (36). Additionally, pharmacological induction of Mfn-2 with Leflunomide increased mitochondrial fusion, with decreased ATP production and tumor growth in pancreatic ductal adenocarcinoma (37). Anti-mitochondrial therapy is a potential approach to target caner progression and metastasis. By inhibiting cancer cell mitochondria, numerous precancerous changes can be targeted. As well as preventing therapy resistance, targeting mitochondria can enhance chemotherapy, radiotherapy, and immunotherapy.

In this Research Topic, we discuss the critical function of mitochondria in cancer cells and TMEs. The mitochondria provide a connection between the cells of the TME and support the survival and progression of cancer cells. By modifying ROS homeostasis, mitochondria control the glycolytic flux and the induction of genes involved in progression and metastasis. Mitochondria in acidic TMEs inhibit immune infiltration, cause immune cell exhaustion, and induce cancer cell immune evasion. In cancer cells, mitochondria provide resistance to chemotherapy, radiotherapy, and immunotherapy. Taken together, mitochondria are crucial targets for drug therapies, and changes in mitochondrial copy number variations and gene expressions function as predictive and prognostic biomarkers.

## Author contributions

PD and VSN equally contributed writing, editing, and proof reading. All authors contributed to the article and approved the submitted version.
